# Supplementary material for: Evolutionary Trajectories of Primary and Metastatic Pancreatic Neuroendocrine Tumors Based on Genomic Variations
Source: Genes (Basel). 2022 Sep 4;13(9):1588. doi: 10.3390/genes13091588 (PMC9498575; doi:10.3390/genes13091588)
Supplement: Supplementary file 1 [file genes-13-01588-s001.zip › Supplementary Figures.pdf]

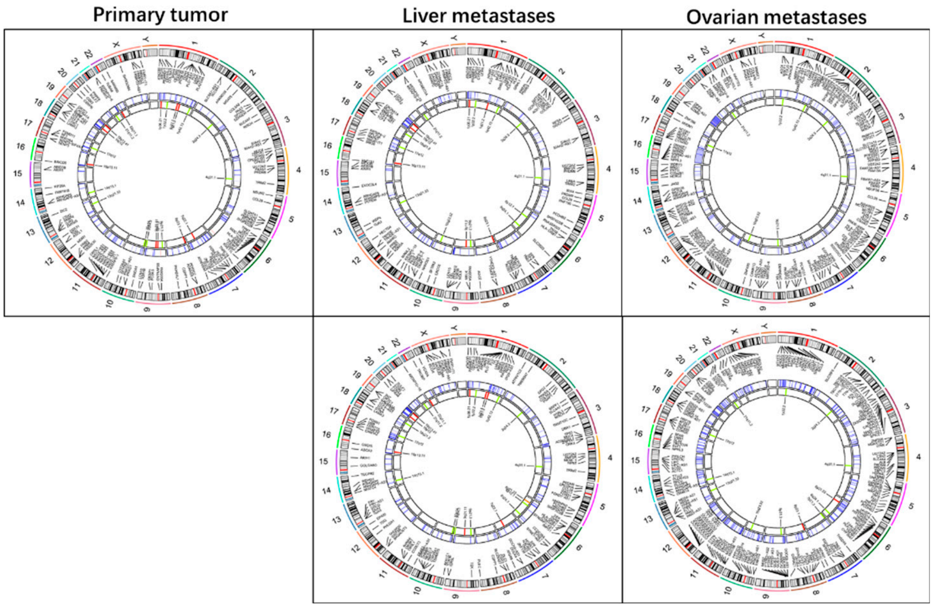

Supplementary Figure S1. High-resolution version of circos plots display the chromosomal distribution of variations in the five tumor samples from the patients.

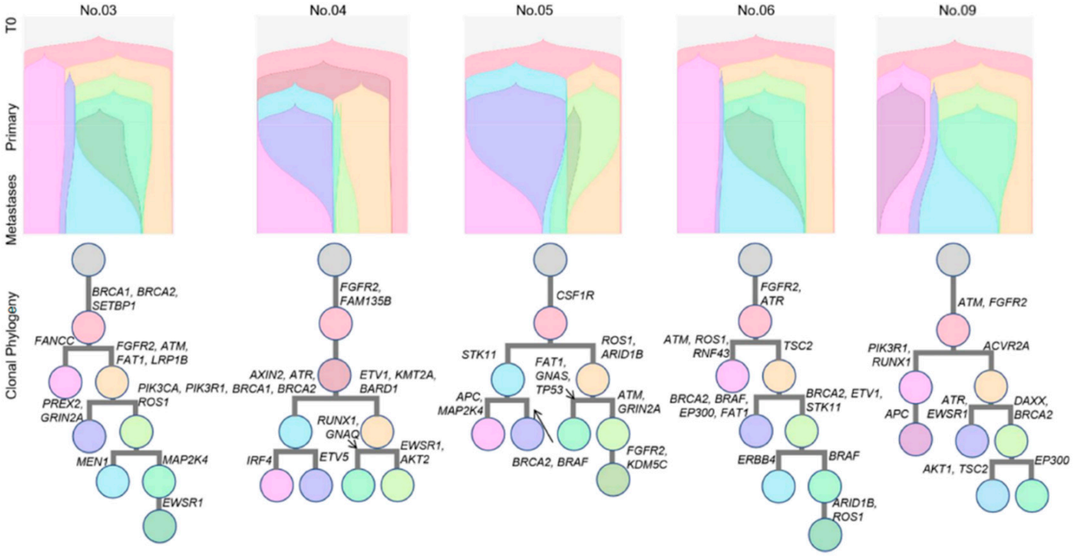

Supplementary Figure S2. Representative cases illustrating distinct patterns of clonal evolution are shown. The fish plot shows the inferred clonal evolution pattern. The phylogenetic trees visualize the estimated order of mutation acquisition. Each node represents featured mutational events, and each circle represents a subclone with cumulative mutational events.
