# Supplementary material for: Evolutionary Trajectories of Primary and Metastatic Pancreatic Neuroendocrine Tumors Based on Genomic Variations
Source: Genes (Basel). 2022 Sep 4;13(9):1588. doi: 10.3390/genes13091588 (PMC9498575; doi:10.3390/genes13091588)
Supplement: Supplementary file 1 [file genes-13-01588-s001.zip › Supplementary Tables.pdf]

Supplementary Table S1. Summary of significantly mutated genes affected by SNPs

Gene	Chrom	HGVS.c	HGVS.p	VariationFunction	Pancreas	Liver
CSK	15	c.1186G>A	p.Val396Ile	missense_variant	Patient7/10	Patient7/10
EPHA2	1	c.1532C>T	p.Thr511Met	missense_variant	Patient1/3	Patient1/3
FLT3	13	c.1073A>T	p.Asp358Val	missense_variant	Patient3/10	Patient3/10
GNAQ	9	c.286A>T	p.Thr96Ser	missense_variant	Patient1/6	Patient2/4/6
		c.303C>A	p.Tyr101*	stop_gained	Patient6	Patient2/4/6
KMT2C	7	c.5053G>T	p.Ala1685Ser	missense_variant	Patient4/11	Patient4/7/9/11(L+R)
KMT2D	12	c.13450C>T	p.Arg4484*	stop_gained	Patient2/5	Patient2
LIMK1	7	c.659G>C	p.Gly220Ala	missense_variant	Patient1/6/9	Patient1/6/9
MEN1	11	c.249_252delGTCT	p.Ile85fs	frameshift_variant	Patient1/3	Patient1/3
NRG3	10	c.1414A>C	p.Ser472Arg	missense_variant	Patient6/11	Patient6/11(L+R)
RANBP2	2	c.2339C>T	p.Pro780Leu	missense_variant	Patient7/10	Patient7/10
ROS1	6	c.5326G>C	p.Asp1776His	missense_variant	Patient1	Patient1/7
TNK2	3	c.484C>T	p.Arg162Trp	missense_variant	Patient1/7	Patient1/7
SETD2	3	c.578C>T	p.Pro193Leu	missense_variant	Patient3/6	Patient3/6

Supplementary Table S2. Copy Number Variations of PanNETs and liver metastases

Gene	Chrom	Type	Copy Number	Patients	Pancreas	livers
BRCA1	17	Gain	3 copy	5 (45.5%)	4 (36.4%)	5(41.7%)
BRCA2	13	Gain	3/4/5 copy	4 (36.4%)	3 (27.3%)	3 (25%)
RANBP2	2	Gain	3/4/5 copy	2 (18.2%)	2 (18.2%)	2(16.7%)
SPTA1	1	Gain	3 copy	1 (9.1%)	1 (9.1%)	1(8.3%)
		Loss	1 copy	1 (9.1%)	0(0)	1(8.3%)
ATRX	X	Gain	3 copy	1 (9.1%)	0(0)	2(16.6%)
ATM	11	Gain	3 copy	1 (9.1%)	1 (9.1%)	1(8.3%)
LRP1B	2	Loss	1 copy	1 (9.1%)	0(0)	1(8.3%)

Supplementary Table S3. Structural variants profiles of PanNETs and liver metastases.

Gene	Type	Chrom	Breakpoint	Patients	Pancreas	livers	Annotation
MITF	Del	Chr3<br>Chr3	70013474<br>70013888	4 (36.4%)	2 (18.2%)	4 (33.3%)	Intron_Variant
a*	Del	Chr6<br>Chr6	31239593<br>31324683	1 (9.1%)	1 (9.1%)	2 (16.7%)	Feature_Ablation
DAXX	Del	Chr6<br>Chr6	33288434<br>33288696	1 (9.1%)	1 (9.1%)	0 (0)	b*
MORF4L1	Tran	Chr15<br>Chr12	79118722<br>68968835	1 (9.1%)	1 (9.1%)	0 (0)	Transcript_Ablation
POLE	Tran	Chr12<br>Chr1	133225595<br>184048732	1 (9.1%)	1 (9.1%)	0 (0)	Transcript_Ablation
CHID1&FLT4	Tran	Chr11<br>Chr5	890461<br>180052847	1 (9.1%)	1 (9.1%)	0 (0)	Bidirectional_Gene_Fusi<br>on
ERBB2	Tran	Chr17<br>Chr9	37871479<br>125428255	1 (9.1%)	0 (0)	1 (8.3%)	Transcript_Ablation
HLA-A&HLA-C	Inv	Chr6<br>Chr6	29910563<br>31239615	1 (9.1%)	0 (0)	1 (8.3%)	Gene_Fusion

a*: HLA-B & HLA-C & RPL3P2 & USP8P1 & WASF5P & XXbac-BPG248L24.10 & XXbac-BPG248L24.12 & XXbac-BPG248L24.13

b*: frameshift_variant& splice_donor_variant& splice_region_variant& intron_variant

Supplementary Table S4. Clinical characteristics and process of a patient (No. 11) with pancreatic neuroendocrine tumor and liver and ovarian metastases

	Location	Size(cm)	Grade	Ki67	Operation		PFS	Lymph node	Nerve invasion	Vascular tumor emboli
					Surgery	Date				
**PanNET**	Head	6.0*5.0*3.5	G2	10-20%	Pancreatectomy	02, 2014	24 M	0/29	-	-
**Liver metastases**	Bilateral liver	3.0*2.5*2.0<br>4.0*3.0	G3	35%	Debulking surgery	04, 2016	18 M	/	/	/
**Ovarian metastases**	Bilateral ovaries	13.0*10.1*8.3<br>8.1*7.6*5.9	G1	2%	Debulking surgery	07, 2019	/	/	/	/
